# Effects of Brain Parcellation on the Characterization of Topological Deterioration in Alzheimer's Disease

**DOI:** 10.3389/fnagi.2019.00113

**Published:** 2019-05-21

**Authors:** Zhanxiong Wu, Dong Xu, Thomas Potter, Yingchun Zhang, The Alzheimer's Disease Neuroimaging Initiative

**Affiliations:** ^1^School of Electronic Information, Hangzhou Dianzi University, Hangzhou, China; ^2^Department of Biomedical Engineering, University of Houston, Houston, TX, United States; ^3^Zhejiang Key Laboratory of Equipment Electronics, Hangzhou, China

**Keywords:** Alzheimer's disease, mild cognitive impairment, high angular resolution diffusion imaging, structural connectivity network, fiber tracking

## Abstract

Alzheimer's disease (AD) causes the progressive deterioration of neural connections, disrupting structural connectivity (SC) networks within the brain. Graph-based analyses of SC networks have shown that topological properties can reveal the course of AD propagation. Different whole-brain parcellation schemes have been developed to define the nodes of these SC networks, although it remains unclear which scheme can best describe the AD-related deterioration of SC networks. In this study, four whole-brain parcellation schemes with different numbers of parcels were used to define SC network nodes. SC networks were constructed based on high angular resolution diffusion imaging (HARDI) tractography for a mixed cohort that includes 20 normal controls (NC), 20 early mild cognitive impairment (EMCI), 20 late mild cognitive impairment (LMCI), and 20 AD patients, from the Alzheimer's Disease Neuroimaging Initiative. Parcellation schemes investigated in this study include the OASIS-TRT-20 (62 regions), AAL (116 regions), HCP-MMP (180 regions), and Gordon-rsfMRI (333 regions), which have all been widely used for the construction of brain structural or functional connectivity networks. Topological characteristics of the SC networks, including the network strength, global efficiency, clustering coefficient, rich-club, characteristic path length, k-core, rich-club coefficient, and modularity, were fully investigated at the network level. Statistical analyses were performed on these metrics using Kruskal-Wallis tests to examine the group differences that were apparent at different stages of AD progression. Results suggest that the HCP-MMP scheme is the most robust and sensitive to AD progression, while the OASIS-TRT-20 scheme is sensitive to group differences in network strength, global efficiency, k-core, and rich-club coefficient at *k*-levels from 18 and 39. With the exception of the rich-club and modularity coefficients, AAL could not significantly identify group differences on other topological metrics. Further, the Gordon-rsfMRI atlas only significantly differentiates the groups on network strength, characteristic path length, k-core, and rich-club coefficient. Results show that the topological examination of SC networks with different parcellation schemes can provide important complementary AD-related information and thus contribute to a more accurate and earlier diagnosis of AD.

## Introduction

As the leading cause of dementia in elderly adults, Alzheimer's disease (AD) is a progressive neurodegenerative disorder characterized by increasing cognitive and behavioral deficits (Mueller et al., 2005). Preceding AD, the mild cognitive impairment (MCI) phase presents with significant cognitive or behavioral deficits and an increased risk of developing dementia (Winblad et al., 2004; Jessen et al., 2014; Daianu et al., 2015; Mckenna et al., 2016). Understanding the physiological deterioration caused by MCI and AD provides an opportunity to develop future treatments and predict AD onset. Many postmortem histological and *in-vivo* imaging studies have demonstrated widespread white matter (WM) alterations in MCI and AD patients (Brun and Englund, 1986; Rose et al., 2000; Bozzali et al., 2002; Nir et al., 2015). The WM degeneration and neuronal death linked to AD progression then creates abnormal connectivity patterns between anatomically related brain regions (Lo et al., 2010). Specifically, demyelination and axonal degeneration cause drastic reductions in WM volume, which may contribute to alterations in structural connectivity (SC) network efficacy. Therefore, AD-related cognitive and behavioral deficits may be directly linked the disconnection of brain regions (Delbeuck et al., 2003; Sorg et al., 2009; Lo et al., 2010), such that altered SC topological patterns reflect the propagation stage of AD.

High angular resolution diffusion imaging (HARDI) has provided an ability to extensively study brain networks in clinical neuroscience (Nguyen et al., 2018). The recent development of accurate and sophisticated HARDI-based tractography methods has encouraged the exploration of regional connectivity and topological network measures, which can quantify MCI and AD-linked brain changes. Graph theory has been frequently employed to detect SC network differences across normal control (NC), MCI, and AD groups, and a variety of topological measures sensitive to SC network disruption can be computed to reveal how AD affects the human connectome. Particular measures of interest include *k*-core, rich-club efficiency, nodal degree, characteristic path length, clustering coefficient, and global efficiency. To perform statistical analysis on SC networks, Kim et al. presented a multi-resolution analysis framework (Kim et al., 2015), in which a Wavelet representation of each anatomical connection was derived at multiple resolutions to analyze AD-related alterations. In Daianu et al. (2015), rich-club properties at a range of degree thresholds were calculated, and their findings indicated that brain network disruptions occurred predominately in the low-degree (< 16) regions of the connectome in AD. In Daianu et al. (2013b), *k*-core was computed to understand the brain network breakdown caused by AD. In Lo et al. (2010), the alterations of various network properties were examined, indicating that AD patients exhibit shorter path lengths, decreased global efficiency, and reduced nodal efficiency. Yao et al. (2010) explored the characteristics of SC networks in MCI and AD, finding that the MCI groups showed a loss of hub regions in the temporal lobe and altered interregional correlations, and that the topological measures of the MCI SC networks exhibited intermediate values. Most of these findings suggest that AD is related to the disruption of structural connectivity, which is characterized by the loss of rich-club organization and network efficiency. Together, the findings suggest that AD is associated with a disrupted topological organization of SC networks, thus providing structural evidence for abnormalities in the SC network integrity of AD patients.

Graph-based analysis of brain structural networks provides a chance to understand how AD-linked structural connectivity abnormalities underlie the cognitive and behavioral deficits of patients. Specifically, the definition of network nodes is one of the most critical steps in network topological analysis, as it assigns the network structure and density for subsequent assessment. Different whole-brain parcellation schemes have been developed to define network nodes, although the effect that these schemes have on the detection of AD propagation stages remains unknown. Accurate brain parcellation provides a foundation for understanding the functional and structural organization of the human brain. During graph-based analysis of the SC networks derived from HARDI, brain parcellation is a key step for the construction of brain anatomical brain network architecture. This step is not trivial, however; the division of the cortex into different numbers of regions affects the structure of the SC network, such that the resulting topological properties of the generated SC network can be significantly changed by the scale of the chosen parcellation atlas (Proix et al., 2016). Considering that brain parcellation schemes are fundamental to the isolation and selection of brain regions, their application plays an important role in revealing the abnormal topological organization of SC networks in MCI and AD.

Cognitive studies have demonstrated that the cerebral cortex is comprised of distinct cortical areas that are interconnected through WM fibers (Sporns et al., 2004; delEtoile and Adeli, 2017). Network analysis represents cortical regions and their connections as a series of nodes and edges, respectively (Lo et al., 2010). Previous investigation have typically relied on a single type of whole-brain parcellation scheme to construct SC networks, such as the 96-region Harvard-Oxford atlas used in Shao et al. (2012), 113-region Harvard-Oxford atlas used in Zhan et al. (2015a), 162-region IIT3 atlas used in Kim et al. (2015), and 68-region Desikan-Killiany atlas used in Daianu et al. (2013b) and Daianu et al. (2015). Each of these schemes presents a different number of parcels, and the effect this has on AD-related SC topological changes has not comprehensively characterized. In this study, SC networks are constructed for NC, early mild cognitive impairment (EMCI), late mild cognitive impairment (LMCI), and AD subjects based on HARDI tractography to fully characterize the manner in which patterns of SC network topological metrics change based on parcellation schemes. Four different whole-brain parcellation schemes over a range of parcellation scales (62, 116, 180, and 333 regions) were used to define SC network nodes: the OASIS-TRT-20 (62 regions) (Klein and Tourville, 2012), AAL (116 regions) (Tzourio-Mazoyer et al., 2002), HCP-MMP (180 regions) (Glasser et al., 2016), and Gordon-rsfMRI (333 regions) (Gordon et al., 2014). Edges were then estimated through deterministic fiber tracking based on orientation distribution function (ODF) fields, which was derived from HARDI images (Iturria-Medina et al., 2008; Descoteaux et al., 2009; Côté et al., 2013; Yeh et al., 2013; Christiaens et al., 2015). To determine if SC topological characteristics changed with different cortical parcellation schemes as AD progressed, SC network topological assessments were performed on a mixed ADNI cohort of 20 NC, 20 early MCI (EMCI), 20 late MCI (LMCI), and 20 AD subjects. Finally, to explore the influence that different cortical parcellation schemes exert on the graph-based analysis of brain SC networks in AD propagation, Kruskal-Wallis tests were employed to identify group differences in network strength, global efficiency, characterized path length, cluster coefficient, *k*-core, and modularity coefficient. Additionally, linear regression analysis was used to examine the changing trajectories of rich-club coefficients for NC, EMCI, LMCI, and AD groups.

## Materials and Methods

### Data

Data used in the preparation of this article were obtained from the Alzheimer's Disease Neuroimaging Initiative (ADNI) database (adni.loni.usc.edu). The ADNI was launched in 2003 as a public-private partnership, led by Principal Investigator Michael W. Weiner, MD. The primary goal of the ADNI has been to test whether serial magnetic resonance imaging (MRI), positron emission tomography (PET), other biological markers, and clinical and neuropsychological assessment can be combined to measure the progression of mild cognitive impairment (MCI) and early AD (Jack et al., 2008; Risacher et al., 2009; Petersen et al., 2010). In this study, 80 subjects were selected from the ADNI database and arranged into NC, EMCI, LMCI, and AD groups according to their ADNI classification. Table 1 shows the demographics of the participants, including age and gender. All 80 participants underwent whole-brain MRI scanning using 3T GE Medical Systems scanners. The acquisition protocol included a T1-weighted image (acquisition matrix = 256 × 256 × 196, voxel size = 1.05 × 1.05 × 1.2 mm^3^, TR = 6.96 ms, TE = 2.83 ms). Furthermore, the participants were scanned with DWI echo planar imaging (EPI) protocol. Specifically, five images with no diffusion sensitization (b0 images) and 41 images along 41 diffusion directions were acquired (b = 1,000 s/mm^2^) with the following parameters: acquisition matrix = 128 × 128 × 55, voxel size = 2.7 × 2.7 × 2.7 mm^3^, TR = 7,200.0 ms, TE = 56.0 ms.

**Table 1:** Demographics information for ANDI participants, arranged into NC, EMCI, LMCI, and AD groups.

	**NC**	**EMCI**	**LMCI**	**AD**
N	20	20	20	20
Gender	8M/12F	13M/7F	11M/9F	10M/10F
Age range (years)	66–87	62–88	61–85	61–90
Mean age (*SD*)	76.85 (6.67)	77.90 (7.26)	75.60 (5.65)	74.15 (7.94)

### SC Network Construction

We evaluated the influence of cortex parcellation schemes on the topological characterization of AD propagation by systematically varying the number of brain parcellated regions. The cerebral cortex of each subject was parcellated into 62, 116, 180, or 333 regions, according to the parcellation templates of OASIS-TRT-20 (Klein and Tourville, 2012), AAL (Tzourio-Mazoyer et al., 2002), HCP-MMP (Glasser et al., 2016), and Gordon-rsfMRI (Gordon et al., 2014), respectively. These four parcellation templates were all spatially normalized into Montreal Neurological Institute (MNI) space (Fonov et al., 2011), and are visualized from different views in Figure S1. Before tracking, the parcellation labels of these templates were used to segment whole brain into clusters of cortical regions. The parcellation templates were co-registered from MNI space (1 mm^3^) to DWI space via T1-weighted images using a 12-degree-of-freedom transformation matrix, using Freesurfer 6.0.0 and DSI Studio.

**Figure 1:**
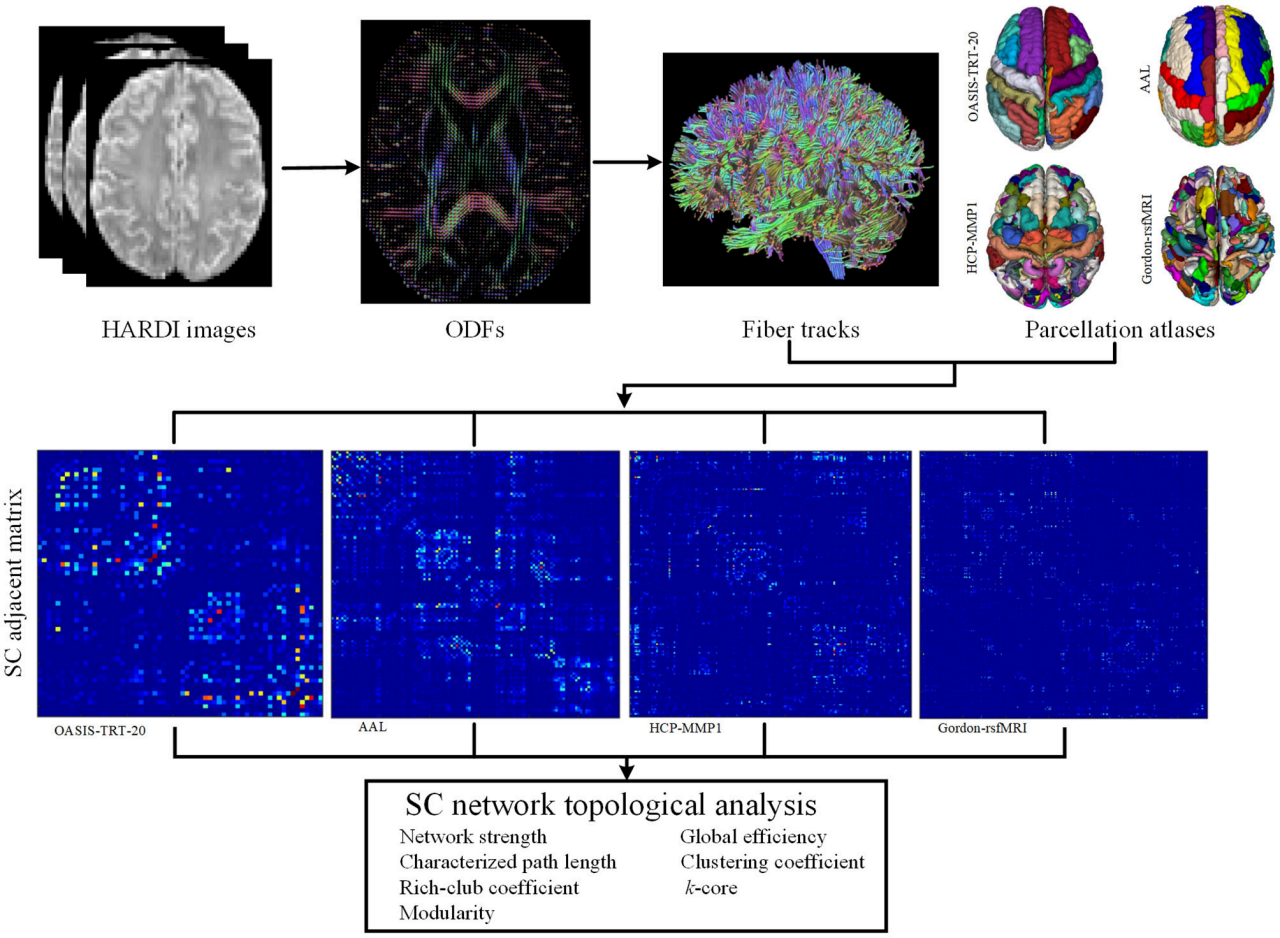
Flowchart of SC network topological analysis. Four parcellation atlases were employed to define the nodes of the SC networks, including OASIS-TRT-20 (62 regions) (Klein and Tourville, 2012), AAL (116 regions) (Tzourio-Mazoyer et al., 2002), HCP-MMP (180 regions) (Glasser et al., 2016), and Gordon-rsfMRI (333 regions) (Gordon et al., 2014). ODFs were computed from HARDI images with a general q-ball imaging (GQI) computational model. Whole-brain tractography was performed using the DSI-Studio tool, and edges were determined by the number of neural connections between each pair of parcellated regions.

The construction of subject-specific SC networks requires a number of complex steps, including cortical parcellation, fiber tractography, and connection strength estimation, as shown in Figure 1. To determine the structural connectivity between each pair of cortical regions, deterministic ODF-based tractography was used. First, the eddy current effects and motion artifacts in the DWI images were corrected using the DiffusionKit toolbox (Xie et al., 2016). DWI images were then denoised using singular value decomposition and non-local means methods, as described in Wu et al. (2018a). Second, a model-free general q-ball imaging (GQI) reconstruction method was employed to estimate ODFs from the HARDI images, with high sensitivity and specificity to WM characteristics and pathology (Yeh and Tseng, 2011). The whole-brain fiber tracking was performed using DSI Studio software (Yeh et al., 2013), with a fractional anisotropy (FA) threshold of 0.2 and a track-turning angular threshold of 60° between each two connections. Cortical connections were established between any set of cortical regions that a fiber bundle passed through or ended in. ODF-based tracking was chosen for this application, as it can resolve multiple fiber populations, including crossing, branching, and merging fibers, and thereby produces more accurate results than DT-based tracking methods (Barnett, 2009; Zhan et al., 2015b; Wu et al., 2018b). The number of reconstructed fibers between different regions were then used to define SC network edges (Hagmann et al., 2007; Houenou et al., 2007; Li et al., 2009), while each parcellated region was regarded as a network node.

### Network Topological Metrics

Graph theory provides a set of measures to concisely quantify the topological properties of brain networks and describe interrelationships between brain regions of interest (ROIs) (represented by nodes in SC networks). Graph-based analysis of brain SC topological patterns allows for the quantification of a broad range of network characteristics. The most common measures used to describe the integrity of healthy or diseased brain networks include network strength, characteristic path length, efficiency, clustering coefficient, *k*-core, rich-club coefficient, and modularity (Sporns, 2010). Topological characterization was performed using the GRETNA (http://www.nitrc.org/projects/gretna/) (Wang et al., 2015) and Brain Connectivity Toolbox (BCT) toolboxes (https://sites.google.com/site/bctnet/) (Rubinov and Sporns, 2010). The utilized network metrics are briefly described below (Cao et al., 2013; Daianu et al., 2013a, 2015).

### Network Strength

For a SC network *G* with *N* nodes and *K* edges, we calculated the strength of *G* as Cao et al. (2013):

(1) $$\begin{array}{r} S_{p}\left(G\right)=\frac{1}{N}\sum_{i\in G}S\left(i\right) \end{array}$$

where *S*(*i*) is the sum of the edge weights linking to node *i*. The strength of a network is the average of the connection strengths across all of the nodes in the network. This metric reflects the extent to which network nodes are connected.

### Clustering Coefficient

The clustering coefficient *C*_*p*_ of a network is the average of the clustering coefficient over all nodes, which indicates the extent of local interconnectivity or cliquishness in a network (Watts and Strogatz, 1998).

(2) $$\begin{array}{r} \text{C}\left(i\right)=\frac{2}{k_{i}\left(k_{i}-1\right)}\sum_{j,k}{\left(w_{ij}w_{jk}w_{ki}\right)}^{1/3} \end{array}$$

Where *k*_*i*_ is the degree of node *i*, and *w* is connection weight. The clustering coefficient will be zero if all nodes are isolated or have just one connection (Watts and Strogatz, 1998).

(3) $$\begin{array}{r} C_{p}=\frac{1}{N}\sum_{i\in G}C\left(i\right) \end{array}$$

### Characteristic Path Length

The path length between any pair of nodes is defined as the sum of the edge lengths along this path. In this study, the length of each edge was assigned by computing the reciprocal of the edge weight, $\frac{1}{w_{ij}}$. The characteristic path length of G was then computed as Cao et al. (2013):

(4) $$\begin{array}{r} L_{c}\left(G\right)=\frac{1}{N\left(N-1\right)}\sum_{i\neq j\in G}L_{ij} \end{array}$$

where *L*_*ij*_ defined as the shortest path between node *i* and node *j*. This metric quantifies the ability for information to be propagated in parallel.

### Network Efficiency

The global efficiency of *G* measures the efficiency of parallel information transfer throughout the network, which can be computed as follows (Cao et al., 2013):

(5) $$\begin{array}{r} E_{glob}\left(G\right)=\frac{1}{N\left(N-1\right)}\sum_{i\neq j\in G}\frac{1}{L_{ij}} \end{array}$$

where *L*_*ij*_ is the shortest path length between nodes *i* and *j* in *G*.

### k-Core Decomposition

To model the basic architecture of SC networks, a *k*-core decomposition algorithm that disentangles the hierarchical structure of the networks was proposed in Daianu et al. (2013b). This *k*-core decomposition outputs a network core that consists of highly and mutually interconnected nodes. This is accomplished by recursively removing nodes with degrees lower than *k*, such that *k* serves as a degree threshold for nodes, ultimately identifying dense subsets of the graph.

### Rich-Club Coefficient

“Rich-club” is a network property that describes how high-degree network nodes are more interconnected than would be expected by chance. The rich-club coefficient is the ratio of the number of connections among nodes of degree *k* (or higher) to the total possible number of connections for those nodes (Daianu et al., 2015). In this study, Rich-club coefficients were calculated at a range of degree thresholds. The rich-club coefficient can be determined as:

(6) $$\begin{array}{r} R\left(k\right)=\frac{E_{>k}}{N_{>k}\left(N_{>k}-1\right)} \end{array}$$

where *R* is rich-club coefficient, *E*_>*k*_ is the number of connections among nodes of degree k or higher, and *N*_>*k*_(*N*_>*k*_−1) is the total possible number of connections if those nodes were fully connected.

### Modularity

Modularity or community structure is a property that is common to brain SC networks, which divides SC network nodes into groups such that structural connections within each group are dense while connections between the groups are sparse. The study of modularity structures in SC networks can provide invaluable help in understanding and visualizing the structure of SC networks. Modularization is an optimization process in which the maximal value of *Q*—the quantity known as modularity—is obtained over all possible divisions of a network (Newman, 2006). Larger *Q* values are indicative of a highly modular network organization, while lower *Q* values indicate a more uniform network structure (Newman and Girvan, 2004). In this study, the *community_louvain* function of the BCT was used to calculate the modularity for the identified SC networks. The employed Louvain optimization is a simple, efficient, and easy-to-implement method for identifying modules in large networks. The optimization comprises two steps. First, the method searches for small modules by optimizing modularity locally. Second, it aggregates the nodes that belong to the same module and builds a new network wherein each node represents a module identified in the first step. These steps are iterated until a maximum of modularity value is attained and a hierarchy of modules is generated (Blondel et al., 2008; Lancichinetti and Fortunato, 2009). Modularity (Q) is defined as:

(7) $$\begin{array}{r} Q=\frac{1}{2m}\underset{i,j}{\sum }\left[w_{ij}-\frac{k_{i}k_{j}}{2m}\right]\delta \left(c_{i},c_{j}\right) \end{array}$$

where *w*_*ij*_ denotes the linking weight between node *i* and node *j*; *k*_*i*_, and *k*_*j*_ are the sums of the weights of the edges attached to nodes *i* and *j*, respectively; *m* is the total link weight in the network overall; and δ(*c*_*i*_, *c*_*j*_) is 1 when nodes *i* and *j* are assigned to the same module and 0 otherwise.

### Topological Metric Estimation

After SC network nodes were determined using four different parcellation schemes, ODF-based tractography was employed to calculate the structural connectivity for each subject. Connection strength values were normalized from [0, 1] and self-connections were excluded. Group average SC matrices were computed for each parcellation scheme. Afterwards, topological measures were estimated using the codes provided in the BCT and GRETNA, including network strength, global efficiency, characterized path length, cluster coefficient, *k*-core, rich-club coefficient, and modularity. Separate from other measures, *k*-core measurement directly reflects how the SC network breaks down as cognitive impairment increases, quantifying how AD affects the human connectome (Daianu et al., 2013b). *k* acts as a degree threshold for network nodes by which *k*-core decomposition creates a subnetwork that consists of highly and mutually interconnected nodes by recursively removing the nodes with degrees lower than *k*. In this study, we used *k*-core analysis to access AD-related anatomical network changes under different whole-brain parcellation schemes including OASIS-TRT-20, AAL, HCP-MMP, and Gordon-rsfMRI. When using a *k* threshold < 17, AD subjects cannot be discriminated from NC and MCI subjects (Daianu et al., 2015). Thus, thresholds of *k* = 20 and *k* = 30 are typically chosen for comparative computations. The rich-club coefficient is the ratio of the number of connections among nodes of degree *k* or higher to the total possible number of connections if those nodes were fully connected (Daianu et al., 2015). This coefficient was computed at a range of *k*-value thresholds from 17 to 39. When the threshold is < 17, the coefficient is close to 1 (Daianu et al., 2015). Modularity optimization is a complete subdivision of the network into non-overlapping modules (Fortunato, 2010), which maximizes the number of within-module edges and minimizes the number of between-module edges. In this study, we used a Louvain community detection algorithm provided in BCT to achieve sub-module decomposition.

### Statistical Analysis

To evaluate discriminating power for AD progressing phases of the network metrices corresponding to different parcellation schemes, statistical analyses were separately performed on each of them using Kruskal-Wallis tests. Additionally, a linear regression model was fitted to rich-club coefficient over a range of *k*-levels from 17 to 39 (Daianu et al., 2015), which was calculated using different whole-brain parcellation schemes. The intercepts and slopes of these regression models generally reflect the associations between rich-club coefficient and progressive AD phases. *P*-values lower than 0.05 were considered statistically significant.

## Results

**Figure 2:**
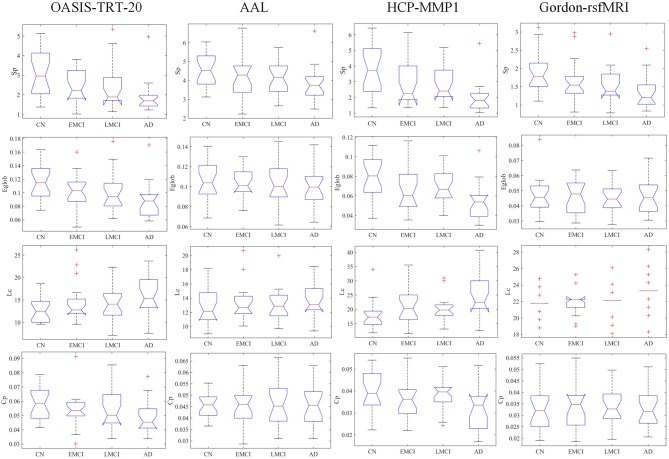
Group median and interquartile ranges of the SC topological metrics for each group, including network strength *S*_*p*_, global efficiency *E*_*glob*_, characterized path length *L*_*c*_, and cluster coefficient *C*_*p*_. *P*-values derived from Kruskal-Wallis tests assessing SC topological differences among NC, EMCI, LMCI, and AD for each parcellation scheme are reported in Table 3.

The group-averaged SC matrices of NC, EMCI, LMCI, and AD groups are depicted in Figure S2. The calculated network metrics (mean ± std) for each parcellation method are listed in Table 2, including network strength *S*_*p*_, global efficiency *E*_*glob*_, characterized path length *L*_*c*_, and cluster coefficient *C*_*p*_. The mean and standard deviation of group network metrics computed for each parcellation scheme are reported in Table 2, with median values and interquartile differences represented in Figure 2. *P*-values derived from Kruskal-Wallis tests assessing the SC differences among NC, EMCI, LMCI, and AD groups for each parcellation scheme are reported in Table 3 (significant values are shown in bold). Significant group differences in *S*_*p*_, *E*_*glob*_, *L*_*c*_, and *C*_*p*_ values were observed when HCP-MMP was used as parcellation scheme (*p* = 0.0007, *p* = 0.0015, *p* = 0.0019, and *p* = 0.0207, respectively). No group differences were found for any AAL-based SC indexes. Significant group differences were found in *S*_*p*_ and *L*_*c*_ (*p* = 0.0032 and *p* = 0.0003, respectively) but not in f *E*_*glob*_ and *C*_*p*_ for SC matrices constructed with Gordon-rsfMRI nodes. For OASIS-TRT-20 parcellation, significant differences in *S*_*p*_ and *E*_*glob*_ were found among the NC, EMCI, LMCI and AD (*p* = 0.0017 and *p* = 0.0080, respectively). No significant group differences in *L*_*c*_ and *C*_*p*_ values were observed for OASIS parcellation.

**Table 2:** The topological metrics (mean ± std) of SC networks in each group, including network strength *S*_*p*_, global efficiency *E*_*glob*_, characterized path length *L*_*c*_, and cluster coefficient *C*_*p*_.

	**CN**				**EMCI**				**LMCI**				**AD**			
	**OASIS**	**AAL**	**HCP**	**Gordon**	**OASIS**	**AAL**	**HCP**	**Gordon**	**OASIS**	**AAL**	**HCP**	**Gordon**	**OASIS**	**AAL**	**HCP**	**Gordon**
*S*_*p*_	3.06 ± 1.14	4.59 ± 0.87	3.80 ± 1.60	1.92 ± 0.55	2.40 ± 0.82	4.31 ± 1.16	2.81 ± 1.36	1.65 ± 0.58	2.39 ± 1.14	4.18 ± 0.91	2.81 ± 1.10	1.50 ± 0.52	1.89 ± 0.81	3.86 ± 0.88	1.95 ± 0.97	1.36 ± 0.45
*E*_*glob*_	0.12 ± 0.03	0.11 ± 0.02	0.08 ± 0.02	0.05 ± 0.01	0.10 ± 0.02	0.10 ± 0.01	0.07 ± 0.02	0.05 ± 0.01	0.10 ± 0.03	0.10 ± 0.02	0.07 ± 0.02	0.05 ± 0.01	0.09 ± 0.03	0.10 ± 0.02	0.05 ± 0.02	0.05 ± 0.01
*L*_*c*_	12.73 ± 2.97	12.75 ± 2.68	17.78 ± 4.96	21.79 ± 1.26	14.44 ± 4.26	13.15 ± 2.33	19.87 ± 4.50	31.89 ± 1.49	14.18 ± 3.85	13.26 ± 2.51	21.01 ± 6.08	21.91 ± 1.67	15.85 ± 4.01	13.92 ± 2.45	24.98 ± 7.41	23.30 ± 2.03
*C*_*p*_	0.06 ± 0.01	0.05 ± 0.01	0.04 ± 0.01	0.03 ± 0.01	0.05 ± 0.01	0.05 ± 0.01	0.04 ± 0.01	0.03 ± 0.01	0.05 ± 0.01	0.05 ± 0.01	0.04 ± 0.01	0.03 ± 0.01	0.05 ± 0.01	0.05 ± 0.01	0.03 ± 0.01	0.03 ± 0.01

*OASIS, AAL, HCP, and Gordon represent OASIS-TRT-20 (62 regions), AAL (116 regions), HCP-MMP (180 regions), and Gordon-rsfMRI (333 regions), respectively*.

**Table 3:** *P*-values of Kruskal-Wallis testing for *S*_*p*_, *E*_*glob*_, *L*_*c*_, and *C*_*p*_ differences among the NC, EMCI, LMCI, and AD groups.

	**OASIS**	**AAL**	**HCP**	**Gordon**
*S*_*p*_	**0.0017**	0.0782	**0.0007**	**0.0032**
*E*_*glob*_	**0.0080**	0.8482	**0.0015**	0.9816
*L*_*c*_	0.0637	0.3627	**0.0019**	**0.0003**
*C*_*p*_	0.0514	0.9988	**0.0207**	0.9538

*The significant p-values are shown in bold. OASIS, AAL, HCP, and Gordon represent OASIS-TRT-20 (62 regions), AAL (116 regions), HCP-MMP (180 regions), and Gordon-rsfMRI (333 regions), respectively. S_p_ denotes network strength. E_glob_ is global network efficiency. L_c_ is characterized path length. C_p_ is clustering coefficient*.

**Figure 3:**
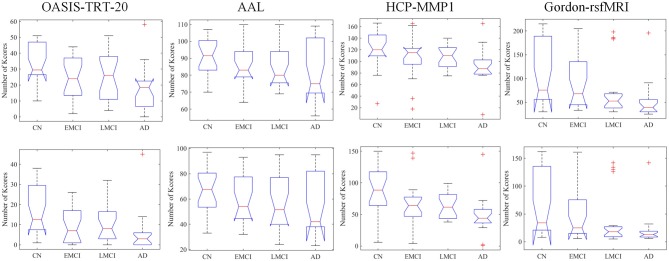
Group median and interquartile ranges of *k*-core network nodes for each group, typically at the thresholds of *k* = 20 and *k* = 30. *P*-values derived from Kruskal-Wallis tests assessing SC topological differences among NC, EMCI, LMCI, and AD for each parcellation scheme are reported in Table 4.

At the typical thresholds of *k* = 20 and *k* = 30, Kruskal-Wallis tests were performed on the number of *k*-core network nodes of NC, EMCI, LMCI, and AD groups. Figure 3 shows group median and interquartile ranges, and the corresponding *p*-values are provided in Table 4. The results indicate that significant group differences in terms of *k*-core were detected when OASIS-TRT-20, HCP-MMP, and Gordon-rsfMRI were used as parcellation schemes (*p* = 0.0038/0.0040, *p* = 0.0105/0.0024, and *p* = 0.0014/0.0023, respectively). For AAL parcellation, no significant group differences in *k*-core number were observed (*p* = 0.2345/0.1696).

**Table 4:** *P*-values of Kruskal-Wallis testing for *k*-core differences among the NC, EMCI, LMCI, and AD groups.

	**OASIS**	**AAL**	**HCP**	**Gordon**
*k* = 20	**0.0038**	0.2345	**0.0105**	**0.0014**
*k* = 30	**0.0040**	0.1696	**0.0024**	**0.0023**

*The significant p-values are shown in bold. OASIS, AAL, HCP, and Gordon represent OASIS-TRT-20 (62 regions), AAL (116 regions), HCP-MMP (180 regions), and Gordon-rsfMRI (333 regions), respectively*.

**Figure 4:**
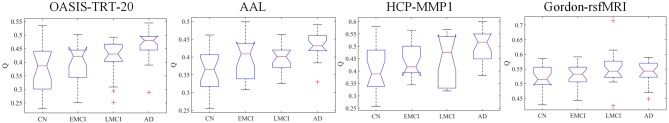
Group median and interquartile ranges of Q coefficient of modularity structure for each group. *P*-values derived from Kruskal-Wallis tests assessing SC topological differences among NC, EMCI, LMCI, and AD for each parcellation scheme are reported in Table 5.

The topological metric of Q quantifies the extent to which SC networks may be subdivided into clearly delineated groups. Figure 4 shows group median and interquartile ranges of modularity statistic Q across different parcellation schemes, and Table 5 shows the *p*-values with significant values in bold. Results indicate that significant group differences were detected when OASIS-TRT-20, AAL, and HCP-MMP parcellation schemes were used to define network nodes (*p* = 0.0004, *p* = 0.0008, and *p* = 0.0148, respectively). For Gordon-rsfMRI parcellation, no significant group differences in Q were observed (*p* = 0.1540).

**Table 5:** *P*-values of Kruskal-Wallis testing for modularity differences among the NC, EMCI, LMCI, and AD groups.

	**OASIS**	**AAL**	**HCP**	**Gordon**
Q of modularity	**0.0004**	**0.0008**	**0.0148**	0.1540

*The significant p-values are shown in bold. OASIS, AAL, HCP, and Gordon represent OASIS-TRT-20 (62 regions), AAL (116 regions), HCP-MMP (180 regions), and Gordon-rsfMRI (333 regions), respectively*.

**Figure 5:**
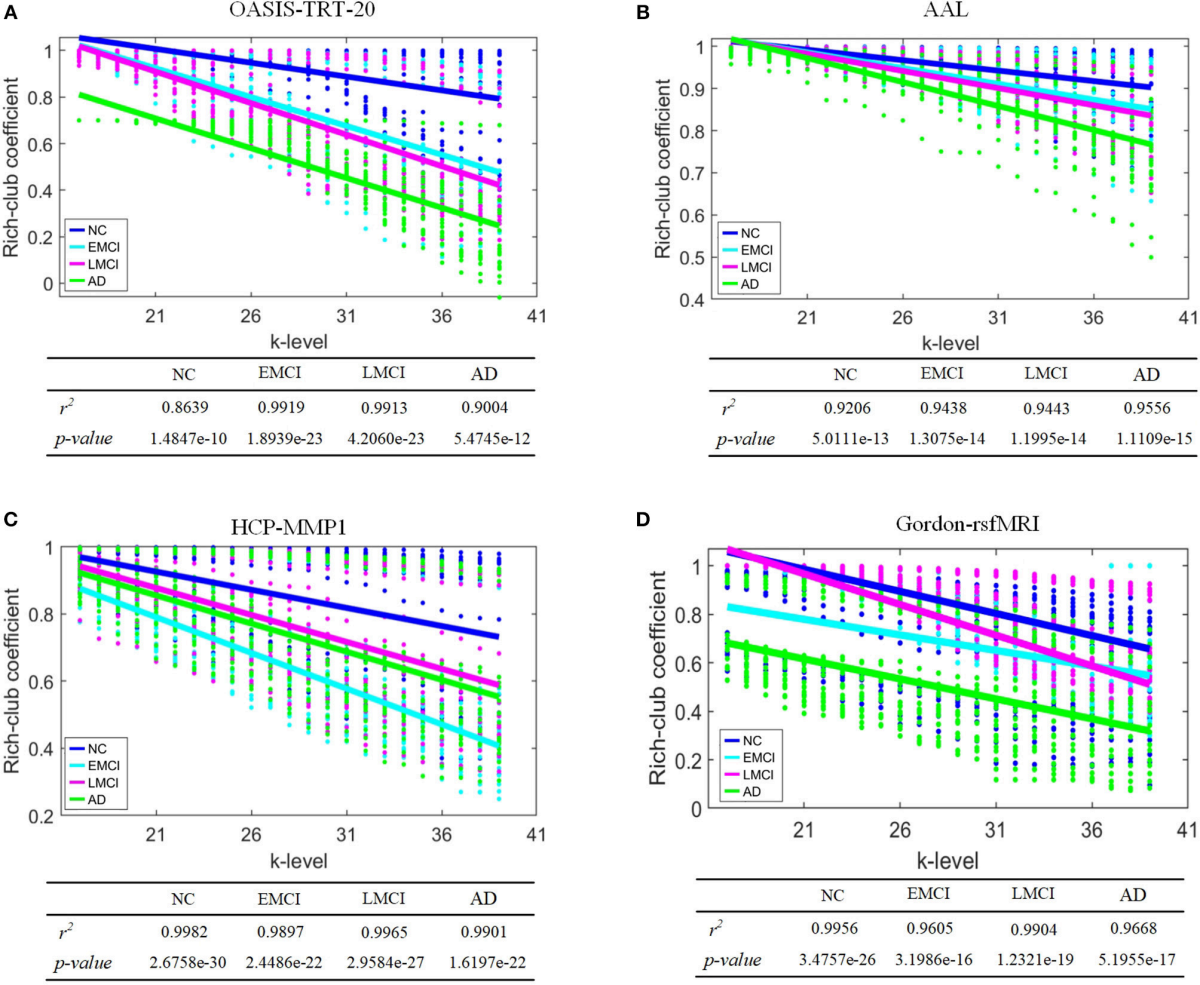
Linear regression of rich-club coefficient over a range of *k*-level from 17 to 39. NC, EMCI, LMCI, and AD groups are represented by blue, cyan, magenta, and green colors, respectively. **(A)** OASIS-TRT-20, **(B)** AAL, **(C)** HCP-MMP, and **(D)** Gordon-rsfMRI.

To evaluate discriminatory efficacy of different parcellation schemes on rich-club coefficient, Kruskal-Wallis tests on the rich-club coefficient *R*(*k*) at each of the *k*-levels from 17 to 39 (Daianu et al., 2015) were performed (*p*-values at each *k*-level are shown in Table 6), a linear regression model was fit to *R*(*k*) as it was calculated over the *k*-levels from 17 to 39. The results in Table 6 indicate that the rich-club coefficients computed based on AAL, HCP-MMP, and Gordon-rsfMRI over the *k*-levels from 17 to 39 are significantly sensitive to the group differences across NC, EMCI, LMCI, and AD (the corresponding *p*-values are < 0.05). Except for the rich-club coefficients computed based on the OASIS_TRT_20 atlas at *k*-level = 17 (*p* = 0.0965), the coefficients at *k*-level between 18 and 39 are able to significantly differentiate the groups (the corresponding *p*-values are < 0.05). Figure 5 shows the linear regression fitted results which reflects the changing trend of rich-club coefficient over the *k*-levels from 17 to 39.

**Table 6:** *P*-values of Kruskal-Wallis testing for rich-club coefficient differences among the NC, EMCI, LMCI, and AD groups.

***k*-level**	**OASIS**	**AAL**	**HCP**	**Gordon**
17	0.0965	**0.0002**	**0.0013**	**<0.0001**
18	**0.0045**	**0.0002**	**0.0007**	**<0.0001**
19	**<0.0001**	**0.0002**	**0.0008**	**<0.0001**
20	**<0.0001**	**<0.0001**	**0.0006**	**<0.0001**
21	**<0.0001**	**0.0002**	**0.0004**	**<0.0001**
22	**<0.0001**	**0.0004**	**0.0005**	**<0.0001**
23	**<0.0001**	**0.0015**	**0.0007**	**<0.0001**
24	**0.0002**	**0.0005**	**0.0004**	**<0.0001**
25	**<0.0001**	**0.0005**	**0.0005**	**<0.0001**
26	**0.0004**	**0.0005**	**0.0003**	**<0.0001**
27	**0.0002**	**0.0002**	**0.0003**	**<0.0001**
28	**0.0001**	**0.0002**	**0.0002**	**<0.0001**
29	**<0.0001**	**0.0001**	**0.0006**	**<0.0001**
30	**<0.0001**	**0.0003**	**0.0009**	**<0.0001**
31	**<0.0001**	**0.0005**	**0.0006**	**<0.0001**
32	**<0.0001**	**0.0005**	**0.0006**	**<0.0001**
33	**<0.0001**	**0.0006**	**0.0007**	**<0.0001**
34	**<0.0001**	**0.0006**	**0.0005**	**0.0002**
35	**<0.0001**	**0.0010**	**0.0006**	**0.0003**
36	**<0.0001**	**0.0007**	**0.0009**	**0.0005**
37	**0.0001**	**0.0008**	**<0.0001**	**0.0008**
38	**<0.0001**	**0.0006**	**0.0008**	**0.0012**
39	**0.0003**	**0.0007**	**0.0012**	**0.0032**

*The significant p-values are shown in bold. OASIS, AAL, HCP, and Gordon represent OASIS-TRT-20 (62 regions), AAL (116 regions), HCP-MMP (180 regions), and Gordon-rsfMRI (333 regions), respectively*.

## Discussion

To this point, evaluating the effects of brain parcellation on the topological characterization of SC networks has been a challenging task, largely due to the lack of universally-accepted parcellation templates that can be used as a reference (Arslan et al., 2017). To provide an effective comparison, this study applied different parcellation schemes and ODF-based tractography to build SC networks for NC, EMCI, LMCI, and AD subjects. Four whole-brain parcellation techniques were used to define the nodes of these SC networks with different number of parcels, and connections were estimated by measuring the pairwise number of neural fiber bundles. To assess the impact of parcellation scheme on the ability to identify differences among NC, EMCI, LMCI, and AD, we explored the topological organization of SC networks. Our findings provide evidence that parcellation schemes have significant impact on topological characterization of brain structural connectivity networks in AD propagation.

After the topological measures were derived from subject-specific adjacency matrices, Kruskal-Wallis tests were employed to investigate their sensitivity to the NC, EMCI, LMCI, and AD groups under each parcellation scheme. Tested measures included network strength, global efficiency, clustering coefficient, characteristic path length, *k*-core, rich-club coefficient, and modularity. We found that these measures were generally sensitive to the selection of parcellation scheme. When interpreting the SC-related results of AD-related studies, the parcellation effect on the calculated measures is a factor that needs to be taken into consideration.

Overall, characteristic path length increased with AD progression in all tested parcellation schemes while network strength, global efficiency, and clustering coefficient decreased, as shown in Table 2 and Figure 2. This is consistent with the results in Lo et al. (2010), Yao et al. (2010), and Daianu et al. (2013a). When the HCP-MMP (180 nodes) parcellation was used to define network nodes, the metrics, including *S*_*p*_, *E*_*glob*_, *L*_*c*_, and *C*_*p*_, displayed significant differences between the NC, EMCI, LMCI, and AD groups. In contrary, AAL atlas cannot discriminate group differences in terms of *S*_*p*_, *E*_*glob*_, *C*_*p*_, *L*_*c*_. The OASIS-TRT-20 scheme was unable to differentiate group differences in terms of *C*_*p*_ and *L*_*c*_, while Gordon_rsfMRI scheme cannot recognize group differences in terms of *E*_*glob*_ and *C*_*p*_. From the results, we could conclude that network strength *S*_*p*_was most robust and sensitive to the characterization of topological deterioration in MCI and AD, while clustering coefficient *C*_*p*_ lacked robustness to whole-brain parcellation atlases. These findings align with a previous study which investigated structural connectivity and the sensitivity of network measures to the parcel number of the parcellation scheme (Zalesky et al., 2010).

*k*-core patterns in the SC networks were then explored, from which the most highly interconnected subnetworks were determined. Kruskal-Wallis test was then performed to determine if *k*-core regions remained intact or were altered by AD progression by eliminating the least reliable anatomical connections (Daianu et al., 2013b). In this study, we analyzed the *k*-core feature at *k* = 20 and *k* = 30, as *k* = 16 has been reported as the minimum value at which the majority of nodes in networks would remain connected. As Daianu et al. (2013b) explored, some *k*-core nodes are lost with AD progression (Figure 3). We used the number of *k*-core nodes as a measure to investigate AD-related network disruption. Significant group differences in the *k*-core patterns of the NC, EMCI, LMCI, and AD groups were found under the OASIS-TRT-20, HCP-MMP, and Gordon_rsfMRI parcellation schemes. Regardless of *k*-level, group difference could not be detected when using AAL atlas.

Modularity was then used to measure the extent to which a network is optimally partitioned into functional subgroups (Rubinov and Sporns, 2011). Due to the breakdown of anatomical connections, the modularity structures of the SC networks exhibited apparent alterations (Figure 4). The breakdown of global informative connections involving the medial prefrontal, posterior parietal, and insular cortices were already apparent in MCI, suggesting that progressive damage to fiber connections begins during the predementia stages of AD (Acosta-Cabronero et al., 2009; Sorg et al., 2009; Sperling et al., 2010; Shao et al., 2012). AD patients then show reduced associative white matter fiber density in the cingulum, the splenium of the corpus callosum, and the superior longitudinal fasciculus (Rose et al., 2000). Coherence studies have further identified disturbed interhemispheric functional connectivity in AD (Brun and Englund, 1986; Wada et al., 1998; Delbeuck et al., 2003). According to Kruskal-Wallis testing, the NC, EMCI, LMCI, and AD groups showed significantly different Q values under most parcellation schemes, with the lone exception of the Gordon_rsfMRI333 parcellation. Further, our results indicate that a loss of *k*-core nodes should increase modularity (Figure 4). This supports the concept that, in addition to mediating internetwork interactions, *k*-core nodes are involved in maintaining the modular structure of functional networks through decreasing network connectivity (Hwang et al., 2017). In accordance with (Daianu et al., 2015), findings here indicate that the breakdown of anatomical connections affected by MCI and AD could increase the modularity coefficient.

Highly connected *k*-core nodes serve as communication hubs, facilitating integrative information processing. These hubs have high nodal degrees and tend to form a rich club—a set of nodes that are densely interconnected. The rich-club coefficient is a related but separate concept from *k*-core, as it evaluates a range of *k*-core thresholds from 17 to 39. The rich-club coefficient is defined as the ratio of the number of connections among nodes of degree *k* or higher to the total possible number of connections if those nodes were fully connected (Daianu et al., 2015). Significant group differences in rich-club coefficient were detected when AAL, HCP-MMP, and Gordon_rsfMRI333 parcellation schemes were used to define SC network nodes. Under the OASIS scheme, no significant group differences were detected at *k*-level = 17. Although some conditional differences were limited, these results help better understand nodal degree alterations in AD. Finally, the changing trends of rich-club coefficients over *k*-level was investigated using linear regression by fitting models with the metrics as predictors for AD propagation. The results indicate that the trends in this metric were different depending on the parcellation scheme used during SC network construction (Figure 5). Overall, rich-club coefficient changes in EMCI, LMCI, and AD accompany a decrease in *k*-core nodes.

From these results, it can be concluded that whole-brain parcellations exert significant influence on the topological characterization of brain structural connectivity networks in AD propagation. Future AD-related structural network studies should attempt to use metrics that are largely robust to the underlying parcellation scheme when attempting to predict AD progression. While it was not possible in this study due to limited available information, the incorporation of clinical and neuropsychological information (such as the Clinical Dementia Rating or Mini-Mental State Examination) should also be considered during analysis. Further, the effect of applied parcellation schemes should be considered during the interpretation of results, as even the most robust measures exhibit some degree of scheme-based variability. As tractography methods could greatly influence the construction of SC networks, a more sophisticated HARDI-based tractography approach may improve the credibility of SC matrices in future.

## Conclusion

Brain parcellation influences the construction of SC network and their topological properties. This work aims to comprehensively explore effect of brain parcellation atlases on characterization of topological deterioration in MCI and AD. There is increasing evidence that widespread network disruptions exist in MCI and AD, and that topological characterization can provide useful biomarkers for the detection of AD progression. In this study ODF-based tractography was employed to construct SC networks from a mixed cohort of 20 NC, 20 EMCI, 20 LMCI, and 20 AD from ADNI under different whole-brain parcellation schemes across multiple spatial scales. The influence of parcellation scheme on the differentiation of the NC, EMCI, LEMCI, and AD groups was then demonstrated. Results suggest differences in the parcellation schemes used to generate SC networks affect the ability for network measures to distinguish structural differences between the NC, EMCI, LMCI, and AD groups. While this study has underlined the importance of the brain parcellation schemes in the SC network analysis of AD progression, further research is required to fully understand the relationship between SC networks and the underlying neural substrates of EMCI, LMCI, and AD at the network level.

## Ethics Statement

In this study, 80 subjects were selected from the Alzheimer's Disease Neuroimaging Initiative (ADNI) database (http://adni.loni.usc.edu/) and arranged into NC, EMCI, LMCI, and AD groups. The ADNI is a comprehensive, multisite longitudinal study, led by principal investigator Michael W. Weiner, M.D., that was launched as a public-private initiative in 2003 to identify the biomarkers that predict MCI and AD progression (Jack et al., 2008; Risacher et al., 2009; Petersen et al., 2010). The primary goal of ADNI is to test whether MRI, positron emission tomography (PET), and clinical/neuropsychological assessment can be combined to measure the progression of MCI and AD. All ADNI subjects gave written informed consent at enrollment for data collection, storage, and use for research.

## Author Contributions

ZW and YZ: study design. ZW: data acquisition. ZW, DX, TP, and YZ: analysis and interpretation, manuscript drafting, and final approval.

### Conflict of Interest Statement

The authors declare that the research was conducted in the absence of any commercial or financial relationships that could be construed as a potential conflict of interest.

## Supplementary Material

The four parcellation templates were all spatially normalized into Montreal Neurological Institute (MNI) space (Fonov et al., 2011), and are visualized from different views (left, right, top, and bottom views) in Figure S1.

Group-averaged SC matrices were obtained by averaging the matrices of all subjects within each group, as shown in Figure S2.

Figure S1 Cerebral cortex parcellation schemes with different numbers of parcels, including OASIS-TRT-20 (62 regions), AAL (116 regions), HCP-MMP (180 regions), and Gordon-rsfMRI (333 regions). The character *M* represents the number of parcellated regions. These schemes are shown from left, right, bottom, and top brain views, respectively. Full index of the parcellated regions can be found in Tzourio-Mazoyer et al. (2002), Klein and Tourville (2012), Gordon et al. (2014), and Glasser et al. (2016).

Figure S2 The group-averaged structural connectivity matrices of NC, EMCI, LMCI, and AD subjects. The strength values were normalized from [0, 1], and self-connections were excluded. From top to bottom, the dimensions of the adjacency matrices are 62 × 62, 116 × 116, 180 × 180, and 333 × 333, respectively.
