# Supplementary figures and images for: Effects of Brain Parcellation on the Characterization of Topological Deterioration in Alzheimer's Disease
Source: Front Aging Neurosci. 2019 May 21;11:113. doi: 10.3389/fnagi.2019.00113 (PMC6536693; doi:10.3389/fnagi.2019.00113)

Fig. S1.


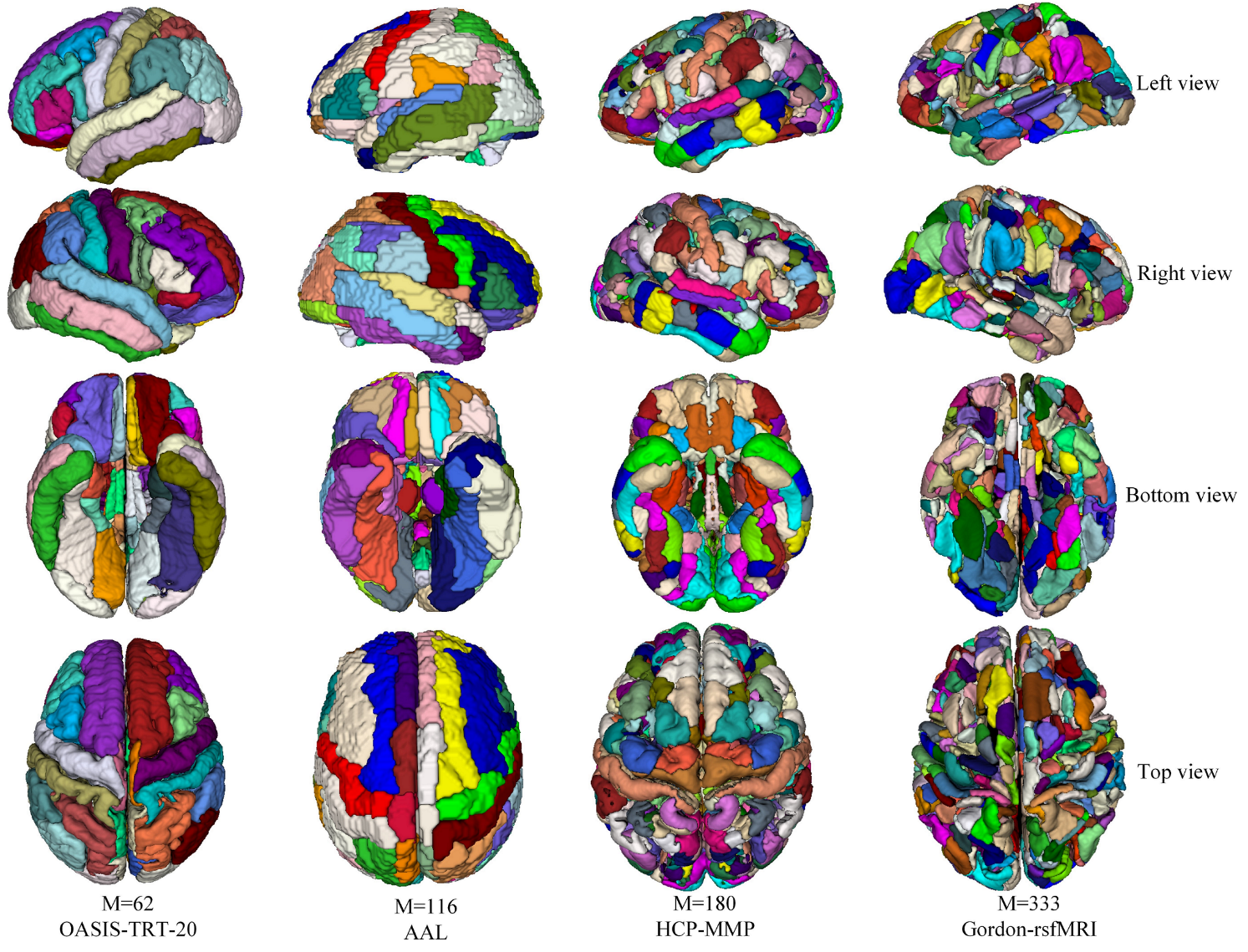


Fig. S2.


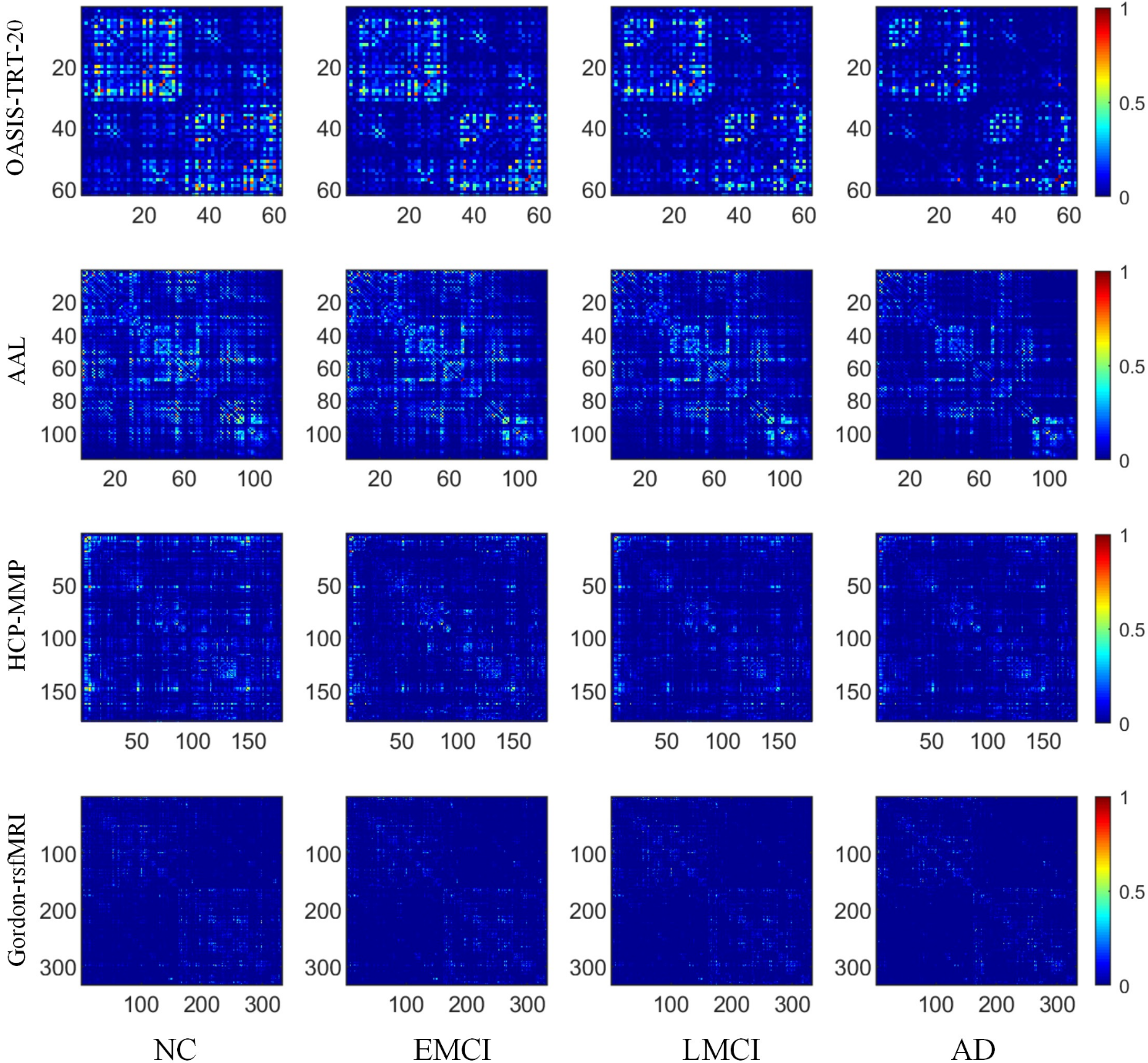

Supplement: Figure S1 — Cerebral cortex parcellation schemes with different numbers of parcels, including OASIS-TRT-20 (62 regions), AAL (116 regions), HCP-MMP (180 regions), and Gordon-rsfMRI (333 regions). The character M represents the number of parcellated regions. These schemes are shown from left, right, bottom, and top brain views, respectively. Full index of the parcellated regions can be found in Tzourio-Mazoyer et al. (2002), Klein and Tourville (2012), Gordon et al. (2014), and Glasser et al. (2016). [file Data_Sheet_1.docx]
